# Glossary

**Published:** 1997

Action potential: A positive charge in a nerve cell’s interior compared with its exterior; caused by the flow of positive *ions* into the cell.
Adenosine: A chemical precursor of adenosine triphosphate, which serves as an energy source in many chemical reactions in the body. Adenosine also modulates the activity of *neurons.*
Agonist: An agent that mimics the actions or effects of another agent (e.g., a drug that mimics the effects of a *neurotransmitter*).
Amino acids: The building blocks of *proteins.* Some amino acids function as *neurotransmitters.*
Amygdala: An almond-shaped structure found within the tip of the temporal lobe of the brain. The amygdala is part of the *limbic system* and has connections to the *hippocampus, septal area, thalamus*, and *hypothalamus.*
Antagonist: An agent that blocks or reverses the actions or effects of another agent (e.g., a drug that blocks the effects of a *neurotransmitter*).
Autonomic nervous system: Nerve cells that regulate involuntary vital functions, such as respiration, the activity of the heart muscles, and the function of glands.
Axon: The long, thin nerve cell fiber that carries integrated electrical information to other *neurons.*
Central nervous system (CNS): The part of the nervous system consisting of the brain and the spinal cord.
Cerebral cortex: The intricately folded outer layer of the *cerebrum*, composed of *neurons.* The cerebral cortex contains areas for processing sensory information and for controlling motor functions, speech, higher cognitive functions, emotions, behavior, and memory.
Cerebrum: The largest portion of the brain; includes the cerebral hemispheres (see *cerebral cortex*).
Cingulate gyrus: An arch-shaped structure closely aligned with the surface of the corpus callosum, which is the bundle of fibers that connects the brain’s two hemispheres.
Concentration gradient: An imbalance in the *ion* concentration on either side of a membrane.
Dendrite: A type of thin, branched nerve cell fiber that extends from a *neuron* to receive information from other neurons.
Depolarization: A process by which the *electrical gradient* along the cell membrane is altered, causing a reduction in the difference between the electrical charges on either side of the membrane (i.e., *membrane potential*).
Dopaminergic: Relating to *neurons* or nerve fibers that release dopamine.
Down-regulation: A decrease in the number or sensitivity of *receptors* as a regulatory mechanism to compensate for their increased activation.
Electrical gradient: The difference in electrical charges on either side of a membrane.
Endorphins: Small *neuropeptides* that bind to *opioid* receptors in the brain and have strong analgesic activity.
Enkephalins: Small *neuropeptides* that bind to *opioid* receptors in many locations in the brain and spinal cord and participate in the regulation of movement, mood, behavior, neuroendocrine regulation, and perception of pain.
Enzyme: A protein that catalyzes (i.e., directs and accelerates) chemical reactions in a cell.
Excitation: An increase in the capacity of a nerve cell to respond to stimuli.
G protein: Intracellular regulatory molecules whose actions are instigated by *neurotransmitters.* Several types of G proteins exist, including stimulatory G proteins (G_s_), which enhance the activities of other *enzymes*, and inhibitory G proteins (G_i_), which inhibit the activities of other enzymes.
GABAergic: Relating to *neurons* or nerve fibers that release gamma-amino-butyric acid (GABA).
Hippocampus: A curved ridge found within the cerebral hemisphere that functions in consolidation of new memories.
Hyperpolarization: A process by which the *electrical gradient* along the cell membrane is altered, causing an increase in the difference between the electrical charges on either side of the membrane (i.e., *membrane potential*).
Hypothalamus: A region of the brain that is involved in basic behavioral and physiological functions. The hypothalamus releases hormones that are critical to the maintenance of the internal environment in response to stress and other stimuli and is implicated in hunger, thirst, and heightened emotional drives.
Inhibition: A reduction in the capacity of a nerve cell to respond to stimuli.
Ion channels: *Proteins* that span the cell membrane, forming pores that regulate the flow of specific charged particles (i.e., *ions*) into and out of the cell.
Ions: Small, electrically charged atoms or molecules.
Limbic system: Parts of the *cerebral cortex, hippocampus, hypothalamus*, and other brain structures that together function in the expression of emotional behavior.
Membrane potential: The difference in electrical charge across the cell membrane.
Neuromodulator: A substance similar to a *neurotransmitter* that is released by a *neuron* and conveys information to adjacent or distant neurons. Neuromodulators indirectly affect the *excitation* or *inhibition* of neurons by changing the way in which neurons react to neurotransmitters.
Neuron: A nerve cell.
Neuropeptide: Molecules composed of short chains of *amino acids*, such as *endorphins* and *enkephalins*, that are found in brain tissues and are thought to act as *neurotransmitters.*
Neurotransmitter: A chemical messenger released by an excited or stimulated *neuron.* After release, neurotransmitters travel across a *synapse* and then bind to a *receptor* on an adjacent neuron, usually triggering a series of chemical and electrical changes in the second cell.
Nucleus accumbens: A brain structure affected by many drugs of abuse and implicated in the rewarding properties of addictive drugs.
Opioid: Any of a group of peptides, such as *endorphins* and *enkephalins*, that bind to or otherwise influence opiate receptors in the brain.
Peripheral nervous system: The nerve cells outside the *central nervous system* that govern motor activity and relay sensory information.
Phosphorylation: A chemical reaction resulting in the addition of phosphate (PO_4_^2−^) groups to other molecules (e.g., *proteins*). Phosphorylation reactions are often critical to regulation of *receptor* activity and the functions of other *proteins*.
Pons: A broad mass of nerve fibers that forms the central portion of the brain stem. The pons participates in control of respiration and coordination of muscular activity.
Postsynaptic cell: The *neuron* to which a *neurotransmitter* or *neuromodulator* binds following release into the *synapse.*
Presynaptic cell: The *neuron* from which a *neurotransmitter* or *neuromodulator* is released into the *synapse.*
Protein: The product of the genetic information encoded in a gene. Proteins are made up of *amino acids; enzymes* are one type of protein.
Protein kinase: An *enzyme* that performs a *phosphorylation* reaction.
Receptor: A *protein* usually found on the surface of a *neuron* or other cell that recognizes and binds to *neurotransmitters* or other chemical messengers.
Reinforcement: The association of a reward with specific behavior, leading to the ability of the reward to elicit that behavior.
Resting potential: The characteristic gradient of electric potential across the membrane of a neuron that is not firing; typically consists of a negative charge on the inside of the cell relative to the outside of the cell.
Second messenger: A signaling molecule that participates in the intracellular reactions resulting when a stimulus, such as a *neurotransmitter*, binds to a *receptor.*
Septal area: A region that stretches as a thin sheet within the cerebral hemisphere and has functional connections with the *hypothalamus* and *hippocampus.*
Striatum: A mass of gray and white brain cells positioned in front of the *thalamus* in each cerebral hemisphere of the brain.
Subunit: A molecule (e.g., a *protein*) that forms part of a larger, more complex molecule.
Sympathetic nervous system: Part of the *autonomic nervous system* that regulates certain involuntary vital functions, especially in response to stress (e.g., accelerates the heart rate and raises blood pressure).
Synapse: A microscopic gap separating adjacent *neurons* where *neurotransmitters* and *receptors* cluster.
Thalamus: A brain region that serves as a communication center and which is involved in the transmission and integration of certain sensations.
Up-regulation: An increase in the number or sensitivity of *receptors* as a regulatory mechanism to compensate for their decreased activation.
Ventral tegmental area: The midbrain region containing dopamine cell bodies that project to various parts of the forebrain, including the *nucleus accumbens.*

## Figures and Tables

**Figure 1:**
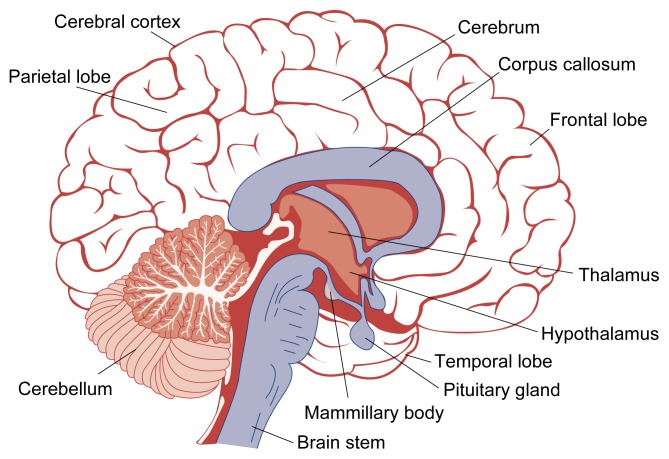
Schematic drawing of the brain.

**Figure 2:**
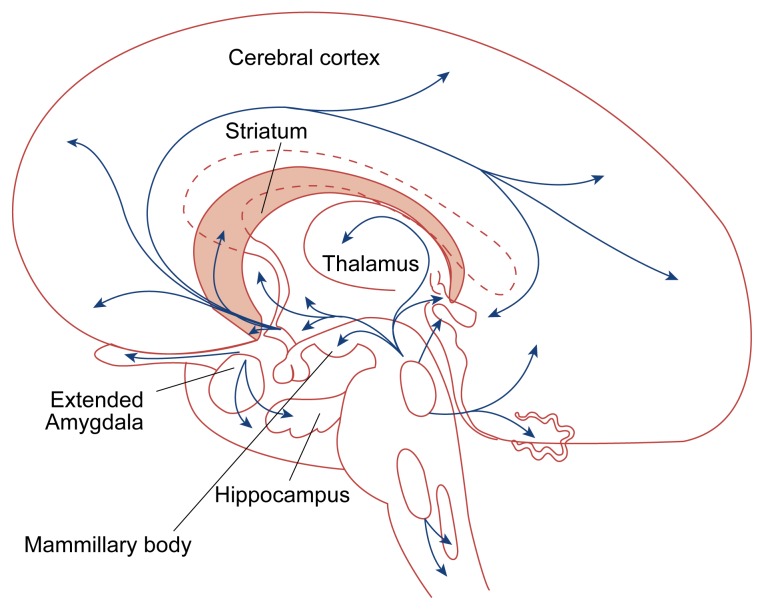
Serotonergic pathways in the brain. The neurotransmitter serotonin appears to exert a widespread influence over arousal, sensory perception, emotion, and higher cognitive functions. Brain cells containing serotonin are located mainly in the midbrain and in the areas immediately above the spinal cord, extending widely to the forebrain, cerebellum, and spinal cord. SOURCE: Adapted from Heimer, L. *The Human Brain and Spinal Cord: Functional Neuroanatomy and Dissection Guide*. 2d ed. New York: Springer-Verlag, 1995.

**Figure 3:**
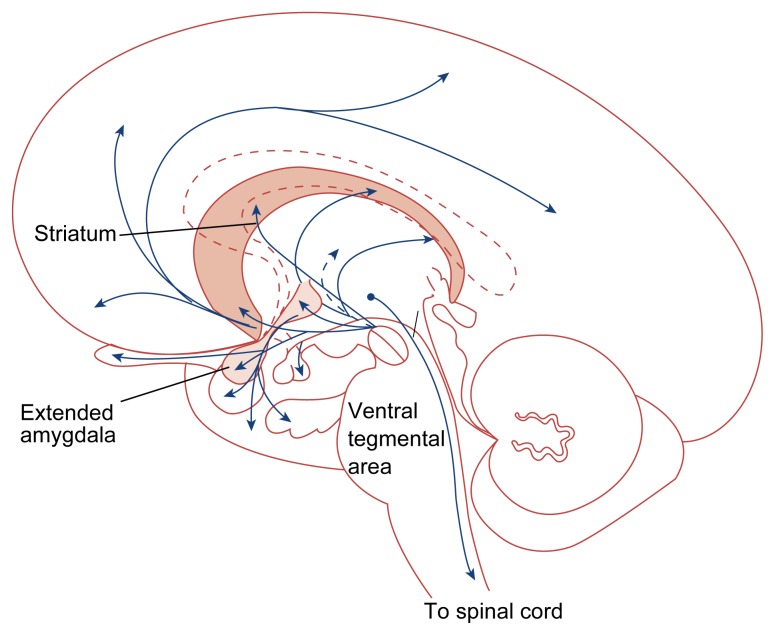
Dopaminergic pathways in the brain. Most dopamine-containing neurons are located within the midbrain, extending to the striatum as well as to various sites in the forebrain. Dopamine modulates such varied functions as emotion, aggression, cognition, the coordination of movement, and aspects of the development of addiction. SOURCE: Adapted from Heimer, L. *The Human Brain and Spinal Cord: Functional Neuroanatomy and Dissection Guide*. 2d ed. New York: Springer-Verlag, 1995.

